# How Will I Be Remembered? Reflection on Virtues in Self-Defining Memories Across Adulthood

**DOI:** 10.1093/geroni/igaa057.1350

**Published:** 2020-12-16

**Authors:** Meghan McDarby, Emily Mroz, Susan Bluck, Brian Carpenter

**Affiliations:** 1 Washington University in St. Louis, Saint Louis, Missouri, United States; 2 University of Florida, Gainesville, Florida, United States

## Abstract

Reflection on memorialization may differentially influence nomination and narration of self-defining memories across the lifespan, including the extent to which positive character strengths (i.e., virtues) are represented. We investigated characteristics of self-defining memories across adulthood and in the context of being memorialized after death. Young, middle-age, and older adults were randomly assigned to narrate a memorialization-based self-defining memory (a memory to describe you after you are gone; n = 103) or current self-defining memory (a memory to describe your current self; n = 99). Participants rated qualities of their memory (e.g., personal significance) and the extent to which the memory represents them as virtuous (e.g., courageous, empathic, etc.). There were no age or condition differences in personal significance of the memory narrative (p = 0.43). However, there was an age-by-condition interaction for representations of virtue described in the self-defining memory, F(2, 199) = 3.94, p = 0.002. Young adults rated their self-defining memories as more virtuous in the memorialization condition than in the current self condition (p = 0.001). Middle-age (p = 0.95) and older (p = 0.94) adults rated their self-defining memories as portraying similar levels of virtue across conditions. Unlike their middle-age and older counterparts, young adults report embodiment of virtue differently in unique contexts. Findings are discussed in the framework of how individuals’ views of the self as virtuous change in relation to time lived and time left to live.

